# 

**Published:** 1894-06-16

**Authors:** 


**Notices of Births, Deaths, and Marriages** are inserted in this column at a charge of 2s. 6d. for an announcement not exceeding four lines.

**Notice to Advertisers and Subscribers.**

All Advertisements, Orders for Copies of The Hospital, and Business Communications should be addressed to **The Publisher, NOT TO THE EDITOR,** The Hospital, 428, Strand, W.C.

The Cost of Subscription, post free, is as follows:—Per week, 2½d.; per quarter, 2s. 8d.; per half-year, 5s. 3d.; per annum, 10s. 6d. Foreign:—Per Annum, 12s. 8d.; payable, in all cases, in advance.

### NOTICE TO CORRESPONDENTS.

All MS., Letters, Books for Review, and other matters intended for the Editor should be addressed The Editor, The Lodge, Porchester Square, London, W.

The Editor cannot undertake to return rejected MS., even when accompanied by stamped directed envelope.

**"Burdett's Hospital Annual," 1894.**

Fifth year of publication. 900 pp. Boards, gilt lettering. A most complete guide to British, Colonial, Indian, and American Hospitals, Dispensaries, Nursing and Convalescent Institutions, and Asylums. Price 5s. Published by The Scientific Press (Limited), 428, Strand, W.C.

**NOTICE.—The Index for Vol. XV. (ending March 31st, 1894) is on sale at the Office of this Journal, price 2½d., post free.**

### Scraps and Gleanings.

The electric light has been introduced into the ophthalmoscopic room at Moorfields, to the advantage of operator and patients.

An inaugural meeting in connection with the Children's Convalescent Home at Felixstowe took place last week. This home will be opened for six months in the year.

The site for the new isolation hospital at Willenhall has been finally decided upon. The building is known as the Casting House. Accommodation will here be afforded for 60 patients.

Mr. Studt has presented the Leominster Cottage Hospital with a donation of £50, which money proceeds from a gigantic "round-about" used at the Leominster May Fair last month.

A subscription list is forthwith to be opened in the North-West of Ireland in support of the projected eye and ear hospital for Derry. The cost of this proposed hospital is about £600.

The annual meeting has just come off in connection with the Drumcondra Hospital, Dublin. A large sum was reported as received in payments from patients, and the finances are generally well sustained.

The Convalescent Home for Women, Marle Hall, near Llandnu, has just been opened in connection with the Birmingham Hospital Saturday movement. The hall, as at present arranged, will accommodate 25 women.

The Oxford Benefit, Friendly, and Trade Societies' Hospital Sunday Fund realised £179 10s. this year. Of the amount subscribed £105 was given to the Radcliffe Infirmary, £25 to the Sarah Acland Home, and the rest divided to the other highly-deserving charities in the city.

The 143rd annual report of the Royal Infirmary, Newcastle, shows that the number of medical in-patients in 1893 decreased, but that there was an increase of 100 surgical in-patients. The income has exceeded the expenditure by £398. Mr. John Taylor has bequeathed £2,000 for special purposes in connection with the infirmary.

### Books Received.

Rivington, Percival, and Co., London.
"A Short Memoir of Emily Minet." Edited by the Rev. C. G. Gepp.

Digby, Long, and Co.
"Three Empresses." By Caroline Gearey.

The Scientific Press.
"The Mother's Help and Guide." By P. Murray Braidwood, M.D.

John Heywood.

"Defects in Plumbing and Drainage Works" Described by Francis Vacher, M.D.

The Isle of Man Steam Packet Company.
"The Little Man Island." By Hall Caine.

David Stott (London).
"How to Tell Your Neighbour's Character." By Paul Bello.

Edward Curtice.
"Curtice's Index to the *Tim s*, the London Morning and Evening Papers, &c., July to September, 1893."

H. K. Lewis.
"Diseases of the Nose and Throat." By F. de Hairland Hall, M.D.

**Periodicals and Pamphlets.**—St. Thomas's Hospital Gazette, The Scalpel, The Guide, The Fireside, Cassell's Magazine, St. Nicholas, The Humanitarian, The Therapist, The Cornhill Magazine, The Service of the King, Guy's Hospital Gazette, The Children's League of Pity Paper, The Zenana, Friendly Greetings, The Cottages and Artisan, Light in the Home, The Child's Companion, Our Little Dots, Reich's Medic inal Anzeiger.

### The Student of Medicine.

### APPOINTMENTS.

F. E. Appleyard, B.A.Cantab., L.R.C.P., M.R.C.S., appointed Clinical Assistant in the Special Department for Diseases of the Throat at St. Thomas's Hospital. Stanwell St. John, M.B., C.M.Edin., M.R.C.S.Eng., appointed House Surgeon to the Stamford, Rutland, and General Infirmary. J. Cagney, M.A., M.D., M.R.C.P., appointed Physician, with charge of in-patients, to the Hospital for Epilepsy and Paralysis. A. M. Collcutt, M.A., M.B., B.C.Cantab., L.R.C.P., M.R.C.S., appointed Resident House-Physician to St. Thomas's Hospital. C. W. Cooke, M.D.Lond., L.R.C.P., M.R.C.S., reappointed Clinical Assistant in the Special Department for Diseases of the Ear at St. Thomas's Hospital. J. Cowper, M.B., C.M.Edin., reappointed Medical Officer of Health for Shanklin District. A. W. Cuff, B.A., M.B., B.C.Cantab., L.R.C.P., M.R.C.S., appointed Assistant House-Surgeon to St. Thomas's Hospital. P. R. W. De Santi, F.R.C.S. Eng., appointed Surgical Registrar to Westminster Hospital. P. C. Fenwick, L.R.C.P., M.R.C.S., appointed Senior Obstetric House-Physician to St. Thomas's Hospital. J. H. Fisher, F.R.C.S., L.R.C.P., reappointed Ophthalmic House-Surgeon to St.Thomas's Hospital. A. S. F. Grunbaum, B.A.Cantab., L.R.C.P., M.R.C.S., appointed Clinical Assistant in the Special Department for Diseases of the Skin at St. Thomas's Hospital. E. M. Hainworth, B.Sc. Lond. L.R.C.P., M.R.C.S., reappointed House-Surgeon to St. Thomas's Hospital. H. R. Hutton, M.A., M.B.Cantab., appointed Physician to the Manchester Hospital for Consumption and Diseases of the Throat and Chest. C. S. Jaffé, M.B., B.S.Lond., L.R.C.P., M.R.C.S., appointed Senior Obstetric House-Physician to St. Thomas's Hospital. N. F. Kendal, M.R.C.S.Eng., L.R.C.P.Lond., appointed Assistant Medical Officer to Woolwich Infirmary. H. R. Kenwood, M.B., C.M.Edin., appointed Medical Officer of Health for Stoke Newington. A. F. W. King, L.R.C.P., M.R.C.S., appointed Clinical Assistant in the Special Department for Diseases of the Throat at St. Thomas's Hospital. A. R. O. Milton, L.R.C.P., M.R.C.S., reappointed House-Surgeon to St. Thomas's Hospital. L. J. Miskin, L.R.C.P., M.R.C.S., reappointed Assistant House-Surgeon to St. Thomas's Hospital. S. G. Morris, M.B., C.M.Edin., appointed Medical Officer for the Cwmtwrch and Cwmllynfell Colliery Districts. J. M. Nicholls, L.R.C.P.Lond., M.R.C.S.Eng., appointed Medical Officer of Health to the St. Ives' Town Council. T. G. Nicholson, B.Sc.Lond., L.R.C.P., M.R.C.S., appointed Clinical Assistant in the Special Department for Diseases of the Skin at St. Thomas's Hospital. R. E. Nix, B.A., M.B., B.C.Cantab., L.R.C.P., M.R.C.S., appointed Resident House-Physician at St. Thomas's Hospital. F. Pershouse, L.R.C.P., M.R.C.S., reappointed Non-resident House-Physician to St. Thomas's Hospital. H. Ramsden, M.B., L.R.C.P.Lond., M.R.C.S.Eng., appointed Deputy Medical Officer for the Saddleworth Rural Sanitary District. A. Ransome, M.D.Cantab., appointed Consulting Physician to the Manchester Hospital for Consumption and Diseases of the Throat and Chest. S. W. F. Richardson, B.Sc.Lond., L.R.C.P., M.R.C.S., reappointed House-Surgeon to St. Thomas's Hospital. J. F. Rudall, M.B., B.Sc.Melb., M.R.C.S., reappointed Ophthalmic House-Surgeon to St. Thomas's Hospital. A. Rudd, M.R.C.S., L.R.C.P.Lond., appointed Medical Officer for the First St. George's District of the Parish of St. Giles, Camberwell. R. F. Symons, L.R.C.P., M.R.C.S., reappointed Clinical Assistant in the Special Department for Diseases of the Ear at St. Thomas's Hospital. G. W. Thompson, B.A., M.B., B.C.Cantab., L.R.C.P., M.R.C.S., reappointed House-Surgeon to St. Thomas's Hospital. C. W. Windsor, M.A., M.B., B.C.Cantab., reappointed Non-Resident House-Physician to St. Thomas's Hospital.

### VACANCIES.

The following vacancies are announced this week, the amount of salary in each case being given after the name of the institution:

**Resident Medical Officers.**

Royal Albert Hospital, Devonport.—Assistant House-Surgeon for six months. Board, lodging, and washing provided. No salary. Applications to Chairman of Medical Committee by June 20th.

**Non-Resident Medical Officers.**

Royal London Ophthalmic Hospital, Moorfields.—Curator, non-resident. Appointment for one year; renewable. Salary £120 per annum. Applications to the Secretary by June 30th.

Tipperary County Infirmary.—Surgeon. Salary £100 per annum. Candidates must be Fellows of the Royal College of Surgeons in Ireland. Applications to Mr. James J. Chadwick, Secretary. Election on June 22nd.

University of Edinburgh.—Chemical Assistant to Professor of Physiology. Salary £180 per annum. Applica ions to the Secretary of the University Court before July 1st.

THE STUDENT OF MEDICINE—*continued.*

### PASS LISTS.

**University of London.**

M.B. PASS EXAMINATION, MAY, 1894.

FIRST DIVISION.—B. Collyer, St. Bartholomew's Hospital; H. B. Dickinson, University College and Royal Infirmary, Liverpool; E. P. A. Mariette, King's College; W. P. Montgomery, Owen's College and Manchester Royal Infirmary; C. T. Parsons, St. Mary's Hospital.

SECOND DIVISION.—E. L. Adams, Guy's Hospital; A. P. Allan, Guy's Hospital; Fanny Armitage, London School of Medicine for Women; J. C. Baker, B.A., St. Bartholomew's Hospital; R. T. Bakewell, University College; J. R. Buckley, Owen's College and Manchester Royal Infirmary; A. T. Collum, Charing Cross Hospital; S. E. Gill, St. Bartholomew's Hospital; J. Harvey, University College and Royal Infirmary, Liverpool; T. W. Hicks, St. Thomas's Hospital; E. Huntley, Guy's Hospital; H. J. Johnson, St. Bartholomew's Hospital; G. L. Kemp, Guy's Hospital; W. A. Marris, Queen's College and General Hospital, Birmingham; A. Miller, Guy's Hospital; E. J. Morgan, St. Mary's Hospital; A. Paling, Middlesex Hospital; L. A. Parry, Guy's Hospital; C. H. Perram, St. Bartholomew's Hospital; A. E. Price, St. Thomas's Hospital; C. M. Rhodes, St. Mary's Hospital; S. S. Swift, School of Medicine and Royal Infirmary, Liverpool; T. S. Vincent, Mason College; H. P. Ward, King's College.

### ACCOUNT BOOKS FOR INSTITUTIONS.

Designed by HENRY C. BURDETT.

Ruled in accordance with the uniform system of accounts advocated by the Metropolitan Hospital Sunday Fund, and prepared for the convenience and assistance of Secretaries who present annual returns for participation in the Hospital Sunday Fund Grants.

These Books are ruled so that the various descriptions of Receipts and Expenditure, &c., may be entered uniformly under the special headings and in the columns prepared for them.

There are TWO SERIES, No. 1 being for the larger Institutions, and No. 2 for Cottage Hospitals and Small Institutions. The sets are sold complete, at a specially reduced price; or each book may be had separately, at the prices set out below.

Full size sample sheets and further particulars will be forwarded to Secretaries and Matrons on application, or specimen volumes will be sent to any Institution willing to pay carriage in the event of business not ensuing. The prices include the name of the Institution being lettered in gilt upon the books.

### SERIES No. 1.—FOR HOSPITALS AND INSTITUTIONS.

(Any of these Books may be purchased separately; a reduction is made if the complete set is taken.)

1. **Analysis Journal.** Royal, 350 pp. Divided into seven sections, with parchment tags. Headings as follows:—
   A. Maintenance.
   1. Provisions. 2. Surgery & Dispensary. 3. Domestic. 4. Establishment Charges. 5. Rent. 6. Salaries. 7. Miscellaneous Expenses.
   B. Administratation.
   1. Management. 2. Finance.
   (Uniform with Headings in Income and Expenditure Table.) Each Heading is ruled with pages containing 15 columns. Price £3.
2. **Cash Book.** Foolscap, 300 pp., printed Headings, specially ruled. Price £1.
3. **Cash Analysis and Receipt Book.** Foolscap, extra width, 300 pp., ruled, with 15 columns, printed Headings identical with Income and Expenditure Table. Price £1 5s.
4. **Secretary's Petty Cash Book.** 300 pp., foolscap, ruled with 13 columns, printed Headings identical with Income and Expenditure Table. Price £1.
5. **List or Register of Annual Subscribers.** Double foolscap, 300 pp., ruled with 10 columns, printed Headings for the years 1893-1900 inclusive. Price £1 15s.
6. **Alphabetical Register Book.** Foolscap, 300 pp., ruled, with date, name and address, and cash columns, cut into alphabetical sections, with totals at end for annual balances. Price £1 6s.
7. **Subscription Register.** Foolscap, ruled with 8 columns, printed Headings for date when subscriptions become due, name and address, and cash columns for years 1893 to 1898 inclusive. Price £1 1s.
8. **Linen Register.** Foolscap long quarto, 300 pp., ruled with columns for Stock, Condemned, Remaining, and Issued Linen, also total in Ward and Remarks, also printed lists of Hospital Linen. Price 8s. 6d.
9. **Income and Expenditure Table.** Basis of uniform system. For yearly balances and statements. Royal. Price 6d. per sheet, or 5s. per dozen.
10. **Special Appeal Account Table.** Foolscap. Price 3d. per sheet, or 2s. 6d. per dozen.

Price of Set Complete, £10 Net.

### SERIES No. 2.—FOR COTTAGE HOSPITALS AND SMALLER INSTITUTIONS.

1. **Cash Analysis, Receipt and Expenditure Book.** Double foolscap, 300 pp., ruled with 18 columns, printed Headings identical with Income and Expenditure Table, specially prepared for Cottage Hospitals and Small Institutions. Price £1 15s.
2. **Secretary's Petty Cash Book.** 300 pp., foolscap, ruled with 13 columns, printed Headings identical with Income and Expenditure Table. Price £1.
3. **Income and Expenditure Table.** Basis of uniform system. For yearly balances and statements. Royal. Price 6d. per sheet, or 5s. per dozen.
4. **Linen Register.** Foolscap, 300 pp., ruled with columns for Stock, Condemned, Remaining, and Issued Linen, also total in Ward and Remarks, also printed lists of Hospital Linen. Price 8s. 6d.
5. **List or Register of Annual Subscribers.** Foolscap. 300 pp., ruled with columns for the years 1893-1900 inclusive. Price £1.
6. **Special Appeal Account Tables.** Foolscap. Price 3d. per sheet, or 2s. 6d. per dozen.

Price of Set Complete, £4 Net.

The books are all uniformly bound in half basil, with gilt letterings, and are ruled on best paper, and in every way prepared for practical use at the offices of Institutions.

### ACCOUNTS, AUDIT, AND TENDERS; the Uniform System of,

For Hospitals and Institutions, with Certain Checks upon Expenditure. Also the Index of Classification. Compiled by a Committee of Hospital Secretaries, and adopted by a General Meeting of the same, 18th January, 1892. And certain Tender and other Forms for securing Economy. By HENRY C. BURDETT. Demy 8vo, cloth, 6s.

This is a hand-book to the account books, explaining their nature and uses, the method of keeping them, and the general principles upon which they were designed.

London: The Scientific Press (Limited). 428, Strand, W.C.

To be suspended in w.c. flush cisterns and used in connection with cooking and cleansing.

A POCKET DISINFECTANT.

### THE NEW DEODORIZER.

Dry, scentless, harmless, universal, perfect.

### 6D. "CONDITAS." 6D.

*Free by Post.* *(Powder in Boxes.)* *Free by Post.*

PURIFIES tainted air, water, fish, meat, game, butter, poultry, cisterns, closets, drains, sewers, utensils, plants, animals, birds. Grand for bath, toilet, mouth-wash, gargle, and breath drink. Perfect hygiene at last for all, automatically.

163, BATTERSEA RISE, LONDON.

